# Supplementary material for: Clocks at a snail pace: biological rhythms in terrestrial gastropods
Source: PeerJ. 2024 Oct 29;12:e18318. doi: 10.7717/peerj.18318 (PMC11529600; doi:10.7717/peerj.18318)
Supplement: Supplemental Information 2 [file peerj-12-18318-s002.pdf]

SUPPLEMENTARY MATERIAL

This is the supplementary material to the article "*Clocks at a snail pace: biological rhythms in terrestrial gastropods*" by Rodrigo B. Salvador & Barbara M. Tomotani.

1. MARINE AND FRESHWATER HETEROBRANCHIA

The Heterobranchia contain diverse marine lineages, alongside some freshwater and terrestrial lineages (the latter, notably include the Hygrophila and the Eupulmonata).

1.1. Marine Heterobranchia

The marine heterobranchs are a diverse group of sea snails and slugs that contain as disparate forms as the sundial snails, nudibranchs, and sea butterflies. As mentioned above, they have been a central piece of chronobiological studies. The first retinal clocks to ever be identified were studied in these animals using the California sea hare (*Aplysia californica*) and the California bubble snail (*Bulla gouldiana*) as model systems (Herzog & Block, 1999). Still, several other heterobranch species (e.g., the ragged sea hare *Bursatella leachii*) have also been studied, albeit not in such detail (Blumenthal et al., 2001; Newcomb et al., 2014). Such studies were part of the groundwork that investigated the anatomical location of the circadian clock in various animals.

Daily endogenous activity rhythms are present in heterobranchs and species can be diurnal (e.g., *Aplysia*, *Bursatella*) or nocturnal (e.g., *Bulla*) (Blumenthal et al., 2001). The eyes of these animals contain a circadian clock and its structure and functioning has been studied in detail (for reviews see Block et al., 1994; Blumenthal et al., 2001; Block & Colwell, 2014).

Nevertheless, animals whose eyes have been surgically removed still synchronize to LD cycles (Blumenthal et al., 2001) (eyeless *Bulla*, however, become diurnal; Block & Davenport, 1982). Non-ocular photoreceptors are present in the ganglia of *Aplysia*, *Bulla* and the nudibranch *Melibe leonina*, and may function as clocks (Roberts & Block, 1982; Block & Lickey, 1973; Blumenthal et al., 2001; Newcomb et al., 2014). In eyeless animals, the rhythms were shown to deteriorate under constant conditions (Blumenthal et al., 2001; Newcomb et al., 2014). Newcomb et al. (2014) thus suggested that, even though both ocular and extraocular photoreceptors would be involved in the synchronization of the clock by light, the former would have the most influence on the expression of circadian rhythms.

Experiments that surgically lesion the cerebral ganglia have pointed that this is the location of the extraocular oscillator (Roberts & Block, 1982), but its precise location and its actual function remain uncertain (Blumenthal et al., 2001). Notably, Block et al. (1974) sowed that red light can synchronize the ocular clock of *Aplysia* even though red wavelengths do not activate the ocular photoreceptors. However, while eyeless animals can be entrained by white light, they cannot do so by red light (Block et al., 1974).

Finally, the circadian clock was shown to modulate the formation of long-term memory in *Aplysia* (see Lyons, 2011 for a review).

Seasonal reproduction in *Aplysia* has been shown to entrained by the photoperiod, with short daylengths stimulating egg laying (Wayne & Block, 1992). Eye removal eliminated that stimulation effect, however, hinting a distinct control of seasonal rhythms from what is seen in daily rhythms (Wayne & Block, 1992).

1.2. Hygrophila

The Hygrophila (previously known as Basommatophora) are a large group of freshwater snails and limpets, closely related to the Eupulmonata and widespread thorough the world. Some species in this clade are models for laboratory studies and are expected to share similarities with terrestrial eupulmonates.

Daily rhythms of locomotion and feeding have been reported for several species of freshwater "pulmonates" and diurnal, crepuscular, and nocturnal patterns have been reported, with diurnal apparently being the most common (Dillon, 2000; Lombardo et al., 2010). These rhythms were shown to be endogenous and persist in constant conditions (e.g., Morgan & Last, 1982; Chaudhry & Morgan, 1983), although for some species (e.g., *Biomphalaria glabrata*, *Lymnaea stagnalis*) there is conflicting evidence of diurnality versus nocturnality and even whether rhythmicity is present at all (e.g., Rotenberg et al., 1989; Stephenson & Lewis, 2011). Those differences could be biological (e.g., distinct populations) or due to methodological issues (e.g., use of laboratory lineages inadvertently selected for certain traits, use of misidentified similar species). *Lymnaea stagnalis* has been used since the 1970s in a variety of studies on hormones, neurobiology, memory, host-parasite interactions, etc. and has become a go-to multipurpose model organism (Fodor et al., 2020; Kuroda & Abe, 2020). However, the issue with its apparent arrhythmicity (Stephenson & Lewis, 2011; Fodor et al., 2020) might have prevented it from becoming a model organism in chronobiology as well. Similarly, *Biomphalaria* spp. (*B. glabrata* in particular) have been model species in medical research as intermediate hosts for schistosomiasis, so most of the research on biological rhythms has focused on the schistosome instead (e.g., Favre et al., 1995, 1997).

Non-ocular photoreceptors have been identified in some Hygrophila species and are considered to play a role in photoentrainment, as snails with surgically ablated eyes still synchronized to the LD cycle (e.g., Kavaliers, 1981; Chono et al., 2002; Ter Maat et al., 2012).

Annual rhythms such as reproduction and migration are synchronized to the seasonal changes in photoperiod. As observed in land snails (see below), studies manipulating the photoperiod have demonstrated that longer light phases can induce growth, sexual maturation, and egg laying in members of the families Lymnaeidae and Planorbidae (e.g., Imhof, 1973; Bohlken & Joosse, 1982; Dogterom et al., 1983; Wayne, 2001; Ter Maat et al., 2007). Shorter light phases are related to an increase in glycogen reserves, tolerance to anoxia, and long-term memory formation in *Lymnaea stagnalis* (Hemminga et al., 1985; Buck et al. 2017; Hussein et al. 2020).

Lundelius & Freeman (1986) showed that distinct populations of the wandering pond snail *Peregriana peregra* differed in their oviposition response being stimulated by either exposure to a long-day (summer) or a short-day (winter) photoperiod. Those authors demonstrated that response is genetically determined and that there is a single gene locus involved in it; the long-day allele, *phi*$^{+}$ (photoperiod interval), is dominant over the short-day one, *phi*. Lundelius & Freeman (1986) also hypothesized that the differences in strategies observed in *Lymnaea stagnalis* (see above) could be the result of the long-day or short-day alleles.

The neural mechanisms of photoperiodism in *Lymnaea stagnalis* were reviewed by Hamanak et al. (2023).

Seasonal migration is also present in freshwater Hygrophila. Species can migrate in the water column (e.g., Shiff, 1966) and some can also migrate to and from the water edge according to the season (e.g., Burla, 1972; Clampitt, 1974). Furthermore, water temperature preference was shown to oscillate seasonally in *Radix auricularia*, which was entrained by the cyclic change in photoperiod (Rossetti et al., 1989).

Aestivation is observed in some species of Hygrophila to survive droughts (Dillon, 2000), but whether this is an immediate response to the environment or a seasonally-programmed stage is still uncertain. In some species, it appears to be a seasonal event linked to natural changes in water level (e.g., Eisenberg, 1966); in the limpet *Ferrissia californica*, however, aestivation is linked to a juvenile stage (e.g., Richardot, 1977).

2. REFERENCES

Block, G. D., & Davenport, P. A. (1982). Circadian rhythm in *Bulla gouldiana*: Role of the eyes in controlling locomotor behavior. Journal of Experimental Zoology, 224, 57—63.

Block GD & Lickey ME. 1973. Extraocular photoreceptors and oscillators can control the circadian rhythm of behavioral activity in *Aplysia*. Journal of Comprative Physiology 84: 367–374.

Block, G. D., Hudson, D. J., & Lickey, M. E. (1974). Extraocular photoreceptors can entrain the circadian oscillator in the eye of *Aplysia*. Journal of Comparative Physiology, 89, 237–249.

Block GD, Geusz M, Khalsa SB, Manivannan K, Michel S, Whitmore D. 1994. Unwinding the snail's clock: cellular analysis of a retinal circadian pacemaker. Netherlands Journal of Zoology 4(3-4): 550–562.

Blumenthal, E. M., Block, G. D. & Eskin, A. (2001). Cellular and molecular analysis of molluscan circadian pacemakers. pp. 371–400 in J. S. Takahashi, Turek, F. W. & Moore, R. Y. (Eds.) Handbook of Behavioral Neurobiology: Circadian Clocks. Vol. 12. New York, Kluwer Academic/Plenum Publishers/Springer.

Bohlken, S., and Joosse, J. (1982). The effect of photoperiod on female reproductive activity and growth of the freshwater pulmonate snail *Lymnaea stagnalis* kept under laboratory breeding conditions. International Journal of Invertebrate Reproduction 4: 213–222.

Buck LT, Bond HC, Malik A. 2017. Assessment of anoxia tolerance and photoperiod dependence of GABAergic polarity in the pond snail *Lymnaea stagnalis*. Comparative Biochemistry and Physiology A 203: 193–200.

Burla, H. 1972. Die Abundanz von *Anodonta*, *Unio pictorum*, *Viviparus ater*, *Lymnaea auricularia* und *Lymnaea ovata* im Zürichsee, in Abhängigkeit von der Wassertiefe und zu verschiedenen Jahreszeiten. Vierteljahresschrift der Naturforschenden Gesellschaft in Zürich 117: 129-151.

Chaudhry, M. A., & E. Morgan. 1983. Circadian variation in the behaviour and physiology of *Bulinus tropicus* (Gastropoda: Pulmonata). Canadian Journal of Zoology 61: 909–914.

Chono, K., Fujito, Y. & Ito, E. 2002. Non-ocular dermal photoreception in the pond snail *Lymnaea stagnalis*. Brain Research 951: 107–112.

Clampitt, P. (1974) Seasonal migration cycle and related movements of the fresh water pulmonate snail, *Physa integra*. American Midland Naturalist 92: 275–300.

Dillon R.T. Jr., 2000. The Ecology of Freshwater Molluscs, Cambridge University Press, Cambridge.

Dogterom, G. E., Bohlken, S., & Joosse, J. (1983). Effect of the photoperiod on the time schedule of egg mass production in *Lymnaea stagnalis*, as induced by ovulation hormone injections. General and Comparative Endocrinology 49(2), 255–260.

Eisenberg, R. (1966) The regulation of density in a natural population of the pond snail, *Lymnaea elodes*. Ecology 47, 889–906.

Favre TC, Bogéa THP, Rotenberg L, Silva HS, Pieri SO. 1995. Cercarial emergence of *Schistosoma mansoni* from *Biomphalaria glabrata* and *Biomphalaria straminea*. Memórias do Instituto Oswaldo Cruz 90(5): 565–567.

Favre TC, Bogéa THP, Rotenberg L, Silva HS, Pieri OS. 1997. Circadian rhythms in the cercarial emergence of *Schistosoma mansoni* by *Biomphalaria tenagophila* at outdoors: a comparative study with *Biomphalaria glabrata.* Biological Rhythm Research 28: 348–357.

Fodor I, Hussein AAA, Benjamin PR, Koene JM, Pirger Z (2020) The unlimited potential of the great pond snail, *Lymnaea stagnalis*. eLife 9: e56962.

Hamanaka Y, Hasebe M, Shiga S. 2023. Neural mechanism of circadian clock-based photoperiodism in insects and snails. Journal of Comparative Physiology A 210: 601–625.

Hemminga, M.A., Koomen, W., Maaskant, J.J., Joosse, J., 1985. Effects of photoperiod and temperature on the glycogen stores in the mantle and the head-foot muscles of the freshwater pulmonate snail *Lymnaea stagnalis*. Comparative Biochemistry and Physiology B 80, 139–143.

Herzog ED, Block GD (1999) Keeping an eye on retinal clocks. Chronobiology International 16(3): 229–247.

Hussein AAA, Baz E-S, Mariën J, Tadros MM, El-Shenawy NS, Koene JM (2020) Effect of photoperiod and light intensity on learning ability and memory formation of the pond snail *Lymnaea stagnalis*. Invertebrate Neuroscience 20: 18.

Imhof, G. 1973. Der Einfluss von Temperatur und Photoperiode auf den Lebenszyklus einiger Süsswasserpulmonaten. Malacologia 14: 393–395.

Kavaliers M. 1981. Circadian and ultradian activity rhythms of a freshwater gastropod, *Helisoma trivolis*: the effects of social factors and eye removal. Behavioral and Neural Biology 32(3): 350–363.

Kuroda R, Abe M. 2020. The pond snail *Lymnaea stagnalis*. EvoDevo 11: 24.

Lombardo P., Miccoli F.P., Giustini M. and Cicolani B., 2010. Diel activity cycles of freshwater gastropods under natural light: patterns and ecological implications. International Journal of Limnology 46: 29–40.

Lundelius JW and Freeman G. (1986). A photoperiod gene regulates vitellogenesis in *Lymnaea peregra* (Mollusca: Gastropoda: Pulmonata). International Journal of Invertebrate Reproduction & Development 10: 201–226.

Morgan E, Last V. 1982. The behaviour of *Bulinus africanus*: a circadian profile. Animal Behaviour 30(2): 557-567.

Richardot, M. 1977. Ecological factors inducing estivation in the freshwater limpet *Ferrissia wautieri* (Basommatophora: Ancylidae). I. Oxygen content, organic matter content and pH of the water. Malacological Review 10: 7–13.

Roberts, M. H. & Block, G. D. (1982). Dissection of circadian organization of *Aplysia* through connective lesions and electrophysiological recording. Journal of Experimental Zoology A 219: 39–50.

Rossetti Y, Rossetti L, and Cabanac M. (1989). Annual oscillation of preferred temperature in the freshwater snail *Lymnaea auricularia*: Effect of light and temperature. Animal Behaviour 37: 897–907.

Rotenberg L, Jurberg P, Pieri OS. 1989. Relationship between light conditions and behavior of the freshwater snail *Biomphalaria glabrata* (Say). Hydrobiologia 174: 111–116.

Shiff, C. (1966) The influence of temperature on the vertical movement of *Bulinus* (*P.*) *globosus* in the laboratory and in the field. South African Journal of Science 62: 210–214.

Stephenson R, Lewis V. 2011. Behavioural evidence for a sleep-like quiescent state in a pulmonate mollusc, *Lymnaea stagnalis* (Linnaeus). The Journal of Experimental Biology 214: 747–756.

Ter Maat A, Pieneman AW, Koene JM. 2012. The effect of light on induced egg laying in the simultaneous hermaphrodite *Lymnaea stagnalis*. Journal of Molluscan Studies 78: 262–267.

Ter Maat A, Zonneveld C, de Visser JAGM, Jansen RF, Montagne-Wajer K, Koene JM. 2007. Food intake, growth, and reproduction as affected by day length and food availability in the pond snail *Lymnaea stagnalis*. American malacological Bulletin 23: 113–120.

Wayne NL and Block GD. (1992). Effects of photoperiod and temperature on egg-laying behavior in a marine mollusk, *Aplysia californica*. Biological Bulletin 182: 8–14.
