# Supplementary figures and images for: Clocks at a snail pace: biological rhythms in terrestrial gastropods
Source: PeerJ. 2024 Oct 29;12:e18318. doi: 10.7717/peerj.18318 (PMC11529600; doi:10.7717/peerj.18318)

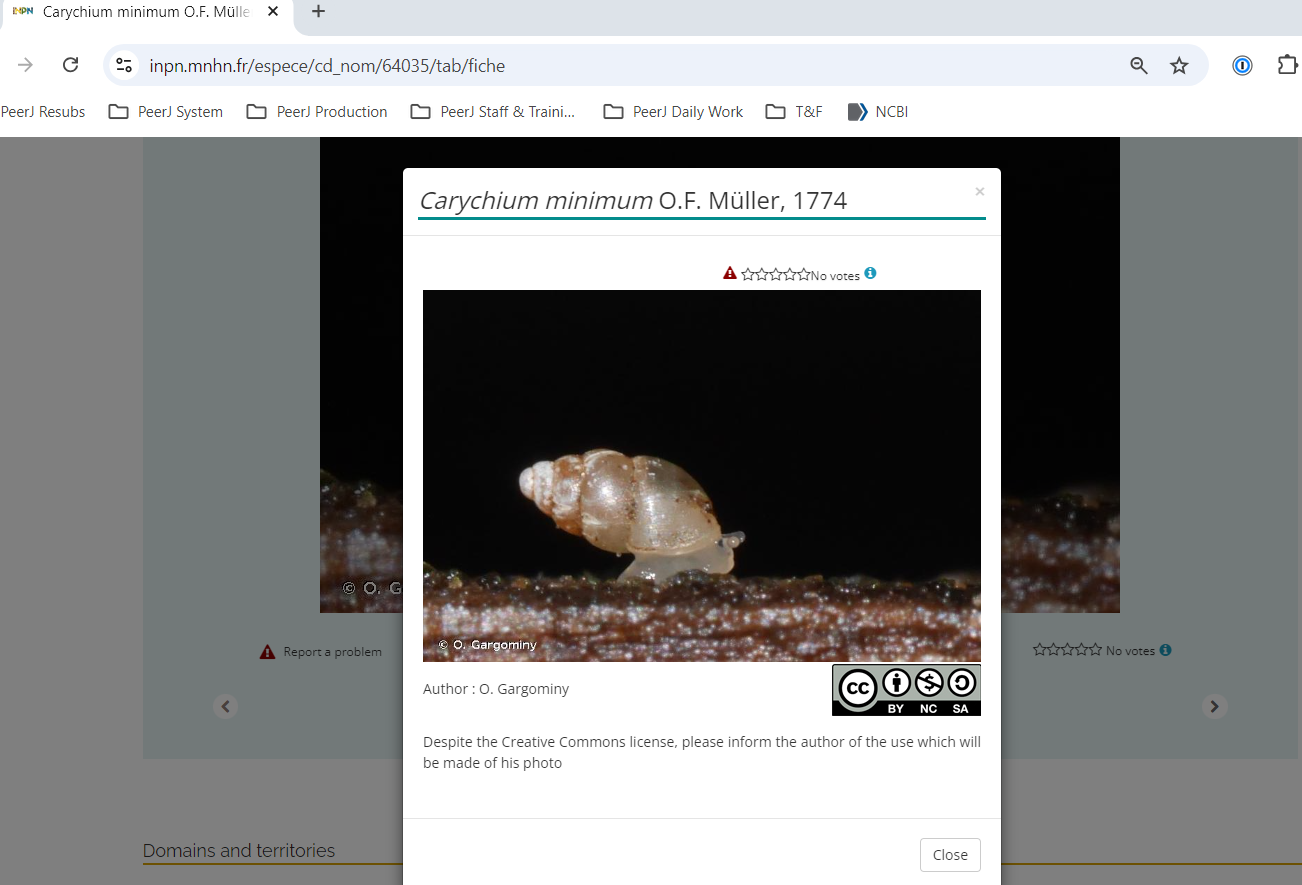

Supplement: Supplemental Information 3 [file peerj-12-18318-s003.png]
